# The effects of webcams on German neonatal intensive care units – study protocol of a randomised crossover trial (Neo-CamCare)

**DOI:** 10.1186/s12913-021-06387-3

**Published:** 2021-05-12

**Authors:** Nadine Scholten, Sebastian Bretthauer, Kerstin Eilermann, Anna Hagemeier, Martin Hellmich, Jan Hoffmann, Dirk Horenkamp-Sonntag, Christiane Jannes, Ludwig Kuntz, Pauline Mantell, Laura Mause, Andreas Müller, Alinda Reimer, Christina Samel, Indra Spiecker genannt Döhmann, Stefanie Wobbe-Ribinski, Christiane Woopen, Till Dresbach

**Affiliations:** 1 University of Cologne, Faculty of Medicine and University Hospital Cologne, Faculty of Human Sciences, Institute for Medical Sociology, Health Services Research, and Rehabilitation Science, Eupener Str., 129 50933 Cologne, Germany; 2 Goethe University, Faculty of Law, Ineges - Institute for European Health Politics and Social Law, Frankfurt/Main, Germany; 3 Department of Business Administration and Health Care Management, University of Cologne, Cologne, Germany; 4 Institute of Medical Statistics and Computational Biology (IMSB), Faculty of Medicine, University of Cologne, Cologne, Germany; 5 Techniker Krankenkasse, Hamburg, Germany; 6 Cologne Center for Ethics, Rights, Economics, and Social Sciences of Health, University of Cologne and Research Unit Ethics, University Hospital Cologne and Faculty of Medicine, Cologne, Germany; 7 Department of Neonatology and Pediatric Intensive Care, Children’s Hospital, University of Bonn, Bonn, Germany; 8 DAK-Gesundheit, Department of Healthcare Research, Hamburg, Germany

**Keywords:** Neonatal intensive care units, Webcams, Preterm, Health services research, Parents, Psychosocial stress, Nurses

## Abstract

**Background:**

The separation of parents and their prematurely born children during care in a neonatal intensive care unit (NICU) can have far-reaching consequences for the well-being of the parents and also of the children. The aim of this study is to evaluate the use of webcams on NICUs and to conduct a systematic assessment of their possible effects on parents and clinical staff. In addition, it aims at determining the need for webcams  in German NICUs and to identify possible barriers and moderators. The development and evaluation of practical guidance for the use of webcams will enable the comprehensive education of clinical staff and parents and, as a result, is intended to mitigate any potential undesirable consequences.

**Methods:**

The study will be based on a mixed methods approach including all groups concerned in the care. Qualitative data will be collected in interviews and focus groups and evaluated using content analysis. The collection of quantitative data will be based on written questionnaires and will aim to assess the status quo as regards the use of webcams on German NICUs and the effects on parents, physicians, and nursing staff. These effects will be assessed in a randomised cross-over design. Four NICUs will be involved in the study and, in total, the parents of 730 premature babies will be invited to take part in the study. The effects on the nursing staff, such as additional workload and interruptions in workflows, will be evaluated on the basis of observation data.

**Discussion:**

This study will be the largest multicentre study known to us that systematically evaluates the use of webcams in neonatal intensive care units. The effects of the  implementation of webcams on both parents and care providers will be considered. The results provide evidence to decide whether to promote the use of webcams on NICUs or not and what to consider when implementing them.

**Trial registration:**

The trial has been registered at the German Clinical Trial Register (DRKS). Number of registration: DRKS00017755, date of registration: 25.09.2019,

## Background

Postnatal care for newborns with special care needs is provided in neonatal intensive care units (NICUs). Newborns with special care needs are usually premature babies, or babies born on time with congenital malformations or complications resulting from childbirth or pregnancy. Premature infants, who make up the majority of the patients cared for in NICUs [1], and very small babies with a birth weight below 1500 g or with a gestational age below the 32nd + 0 week, in particular, often need long-term care in a NICU [2].

During their stay in the NICU, the patients are separated from their parents most of the time. This separation, especially from the mother, can have far-reaching consequences for the mother [3], and a great impact on the bond between the parents and the child [4]. The separation of a mother and her child can impede the development of maternal feelings [5] and decrease the mother’s sense of responsibility and sensitivity [6]. At this time, when role structures are changing, the relationship between fathers and children is playing an increasingly important role. It has been shown that more and more fathers of premature babies are suffering from depression and anxiety [7]. Such psychological strains, which may manifest over the long term, can in turn have an impact on the development of the preterm infant [8]. Furthermore, it has been shown that unrestricted visiting hours and developmentally supportive care can foster parental satisfaction [9]. One way to counter the consequences of the spatial separation between newborns and their parents is to set up webcams in NICUs. Through a webcam installed by the newborn’s bed, parents and other family members can keep in touch with the baby when they are not on site. There are expected to be indirect effects on the medical outcomes for the newborn through, for example, the increased well-being of the parents [10]. The strengthened feeling of being close to the child is understood to promote lactation and thus to foster the nutrition of the newborn with breast milk [11]. In addition to the positive effects of webcams, however, a few parents also report increased fears, triggered by the observation of critical situations as well as by the difficulty of estimating the child’s condition from afar [12].

Besides the positive and negative aspects for the parents and newborns mentioned above, webcams also have an impact on the NICU staff involved in the patient care. It is important to take into account the time it takes to operate the cameras, and the possible additional work caused by a higher number of requests from parents [13]. Nurses associate the implementation of webcams with an increase in workload and stress caused by incoming phone calls from parents [14]. Although some hospitals already offer a webcam system, the use of webcams has not yet been evaluated in a structured manner. The aim of this study is to examine the use of webcams on German NICUs in a quantitative manner by describing the effects on parents and on care providers, and to evaluate the installation of webcams in selected German NICUs. In addition to evaluating the implementation and use of this technology in NICUs, the needs of parents, as well as the willingness of care providers to implement this new technique, will be assessed in a descriptive way. As part of the project, practical guidance for the use of the webcams will be developed and evaluated, with the aim of reaping the benefits and reducing the potential negative consequences of the use of webcams for parents and care providers.

## Method/ design

**Fig. 1:**
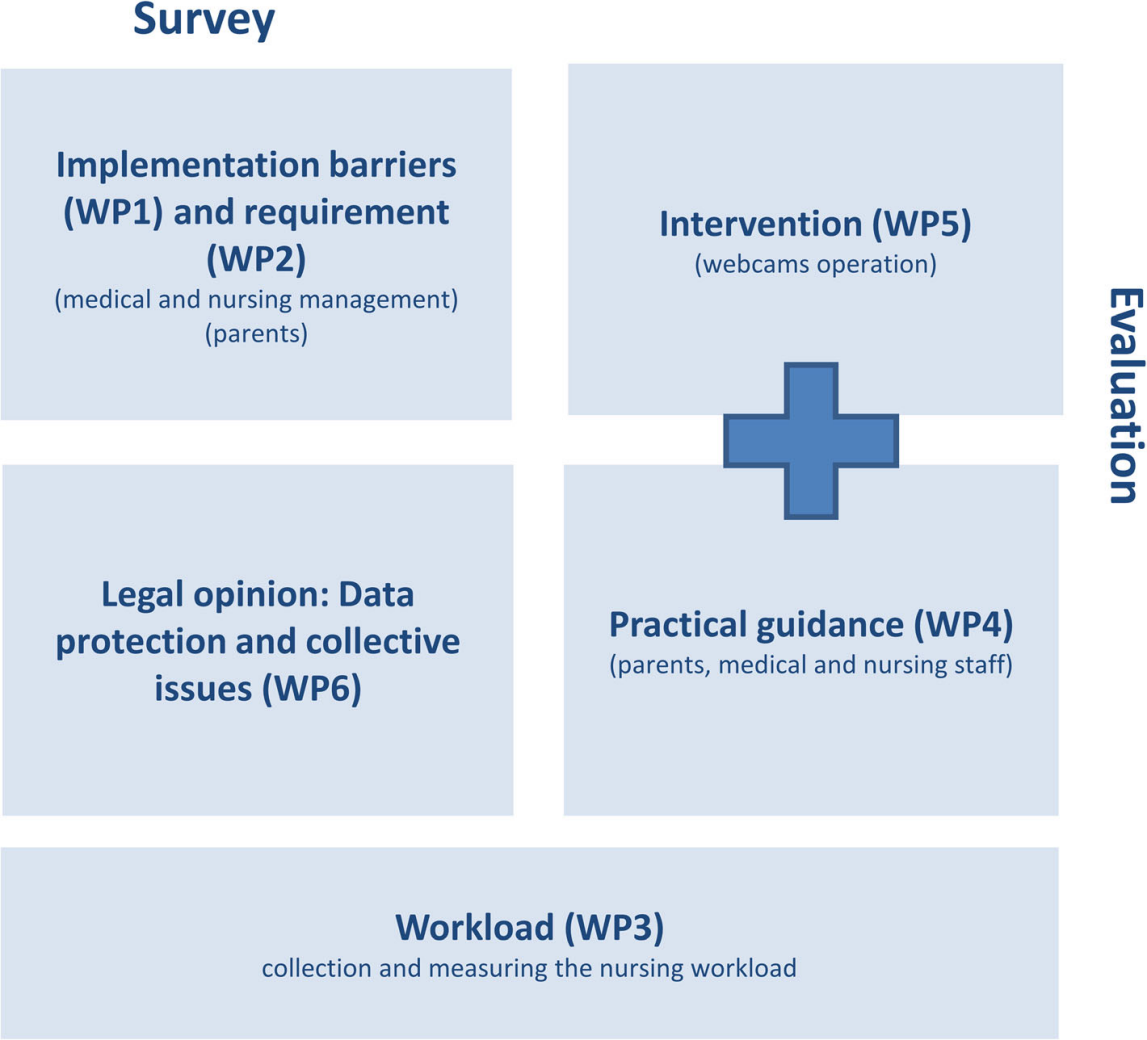
Presentation of the work packages

The study will be conducted with a mixed methods approach, and will include several work packages (WP): (a) qualitative (one-on-one interviews, group discussions and observations (WP1, WP2, WP4, WP5 and WP6)) and (b) quantitative (standardised postal surveys (WP1, WP2 and WP5)) forms of data collection, the collection of observational data on camera-related activities and the time requirements for such activities (WP3) and the evaluation of log-in data (WP5)). Figure 1 presents the different work packages.

### Work package 1

Analysis of implementation barriers. For this purpose, a written survey of the medical and nursing leaders of all NICUs in Germany (*n* = 211) will take place. On the basis of (*n* = 8) qualitative interviews with neonatal nurse (*n* = 4) and physician (n = 4) leaders, a postal questionnaire will be developed. This will aim to capture the attitude towards the use of this technology and to identify possible barriers to its implementation. The ‘Total Design Method’ (TDM) of Dillman [15], with four postal survey waves, will be applied to achieve a high return rate. Data will be managed by the Institute of Medical Sociology, Health Services Research and Rehabilitation Science (IMVR).

### Work package 2

Recording of parental needs. The aim is to capture the attitudes of parents of preterm infants towards the use of this technology. Possible concerns of parents will be raised and addressed both qualitatively and quantitatively. The questionnaire will be developed on the basis of 16 qualitative interviews with parents of premature babies (mothers: n = 8 and fathers: n = 8). The survey documents will be sent by the participating health insurance companies to mothers of preterm infants with a birth weight of less than 1500 g and a current age between 6 and 18 months (expected number to be dispatched *n* = 2100). Data will be managed by the Institute of Medical Sociology, Health Services Research and Rehabilitation Science (IMVR).

### Work package 3

Analysis of the additional workload for nursing staff resulting from the use of webcams in NICUs. Data on the additional workload will be collected in an observational study using a methodology based on those of Langhammer et al. [16] and Sülz et al. [17]. Data on nursing activities and workload will be collected using study nurses who will passively observe the nurses caring for the patients. Using tablet PCs and a self-modified software application, the study nurses will classify the nurses’ activities into different categories (direct care, indirect care, and camera-related activities) and document the time spent on each activity. The documentation of all activities conducted by the nursing staff will allow an assessment to be made of how camera-related activities interrupt the workflow of nurses. Assuming a 30% probability of the occurrence of a camera-related activity, the nurses will have to be observed over a minimum of 300 days in order to obtain a reliable picture of the time spent on the different categories of activities. The days on which nurses are observed will be evenly distributed between the time before and the time after the implementation of the practical guidance, which will be developed in WP4. Besides the observed work intensity, information on the work intensity as perceived by the observed nurses on each day will be collected in a survey using a construct developed by Richter and Richter et al. [18, 19], which has been used in the NICU setting by Sülz et al. [17]. To control for the daily workload in the NICUs when assessing the nurses’ real and perceived work intensity, data on daily bed occupancy and on the case-mix for each day within the study period will be collected for each NICU. Further, characteristics of the NICU, such as the number of beds, physicians, and nurses, will be obtained from the hospitals’ controlling departments. Data will be managed by the department of health care management of the University of Cologne.

### Work package 4

Development and evaluation of practical guidance, which will consist of a basic module and complementary modules specifically adapted to the needs of parents and care providers. The aim of this printed brochure will be to support an informed decision for or against the use of a webcam and to facilitate the use of this new technology. Questions from parents and from health professionals will be addressed. Based on the parents’ experience, the pros and cons will be systematically studied and presented, to allow for an informed decision for or against the use of a webcam. At the same time, it is intended that the practical guidance will identify strategies for dealing with webcams, and thus absorb possible stresses and increase the satisfaction of parents, as well as that of doctors and nurses. The sensitisation of doctors and nurses to the needs and problems of parents arising from the use of the webcam, and the ability to respond to them in a sound manner through the practical guidance, should in the long term help to avoid barriers to implementation by reducing reservations. The practical guidance will be based on an ethnographic observational study [20, 21]. The observational study is carried out on the basis of a flexible observation guide and field notes are produced according to a predefined scheme. This will be followed by a group discussion procedure with pairs of parents to collect further information on their experiences, wishes and problems in dealing with webcams on NICUs and to (further) develop the interview guides for the interviews that will follow. The interviews will be guideline-based, semi-structured qualitative interviews with parents using a webcam (*n* = 20) as well as nurses (*n* = 10) and doctors (*n* = 5) working on a ward that already has webcams set up or where webcams are currently being implemented. In conclusion, based on the knowledge gained from the observations, group discussions and interviews, a modular system of individual hand-outs (practical guidance) will be created. This will be evaluated in work package 5. Data will be managed by the Research Centre Ethics affiliated to University Hospital in Cologne.

### Work package 5

**Fig. 2:**
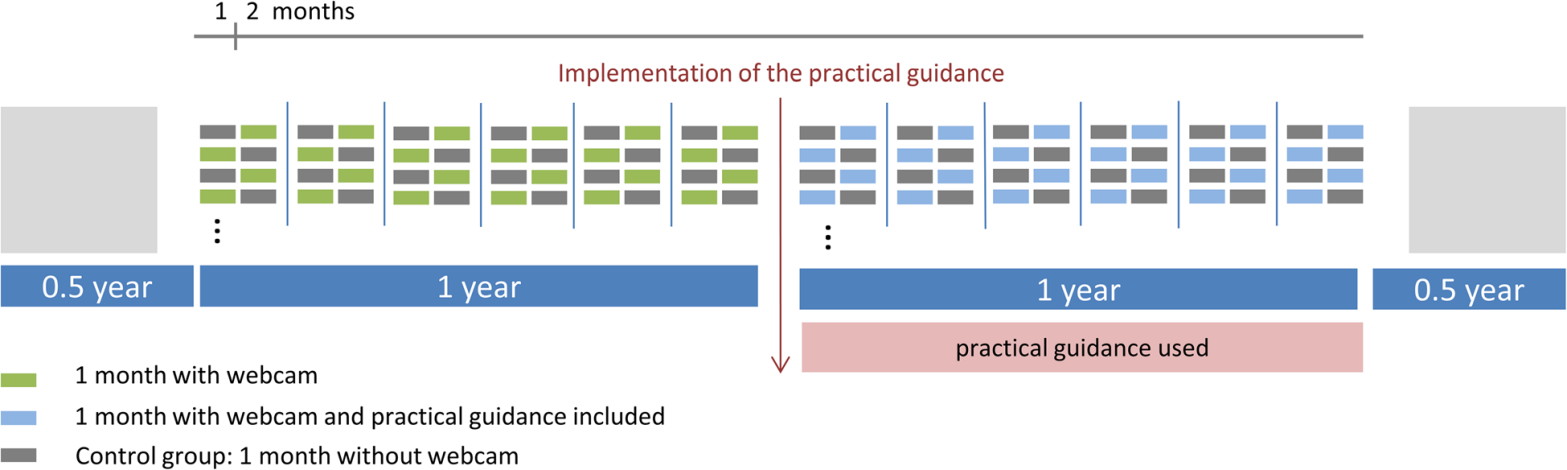
Schematic overview of intervention study

**Fig. 3:**
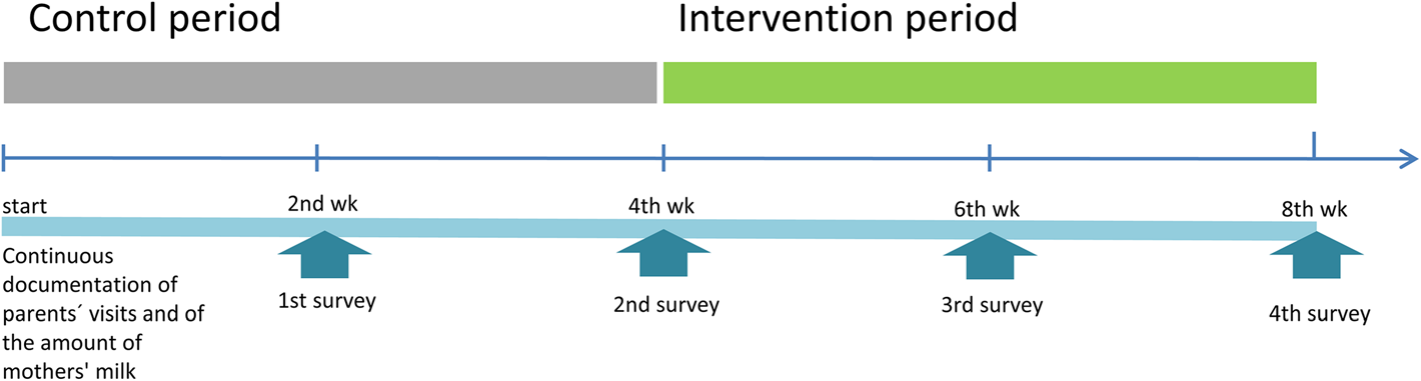
Intervention timescale

The evaluation of the use of webcams will be based on the Medical Research Council (MRC) framework for the evaluation of complex interventions [22]. The intervention is to be regarded as complex according to the MRC framework, as a variety of outcomes have to be considered and different parties will be affected by the intervention, which will take place in a complex organisational setting. The evaluation of this complex intervention will be carried out as part of a randomised cross-over design trial using a waiting-list control group design. The evaluation of the outcomes (summative, ex-post) will be accompanied by a process evaluation (formative, ongoing).  A schematic overview of intervention study can be found in Fig. 2. In four cooperating NICUs (University Hospital Cologne, University Hospital Bonn, University Hospital Düsseldorf, Marienhaus Klinikum St. Elisabeth Neuwied) all parents with a premature baby below 1500 g (*n* = 730) will be invited to take part in the study by the treating neonatologist. Parental consent will be obtained for participation in the study. If the parents refuse to participate in the study, they will be asked to fill out a short questionnaire, which will inquire about the possible reasons for refusal. After the participating parents have been included in the study they will be randomly allocated to the control group or the intervention group by pre-prepared study documents, which randomise the participants to the different points in time of the intervention. Allocation to the control or intervention group is based on a pre computer generated randomisation list for each NICU created by a statistician. Allocation concealment will be guaranteed by opaque, sealed envelopes. After a month, the control group will change with the intervention group. Thus, both groups will receive the intervention, but with a time delay of 4 weeks. The first group will receive the camera in the first 4 weeks of the study period, whereas the second group will receive the camera in the last 4 weeks of the study period. The intervention timescale is presented in Fig. 3.

The randomised crossover design was chosen because blinding of the control group is not possible and thus contamination effects are to be expected. Because of the big differences between the participating clinics with regard to organisational structures, and therefore the expected differences in the outcomes, cluster randomisation is not possible.

The survey will focus on psychosocial factors related to parent satisfaction and psychosocial distress (primary outcome) and possible effects on visit frequency, breastfeeding behaviour and breast milk production (secondary outcome). In addition to the quantitative analysis of the parents’ survey outcomes, there will be an analysis of the log-in data from the webcam portal. By linking these data with the survey data, the different usage behaviours can be descriptively explored, and possible connections to the outcomes can be derived. In the second year of the evaluation, all intervention groups will receive the practical guidance. Data will be managed by the Institute of Medical Sociology, Health Services Research and Rehabilitation Science (IMVR). The pseudonymised data is administered by a data manager who operates technically and organisationally separated from the evaluating scientists. The primary outcome will be analysed after completion of data collection, no interim analyses are planned. All adverse events caused by the camera system will be collected and reported.

Sample size calculation was derived from case numbers of premature neonates with a birth weight below 1500 g of each participating clinic. There has been no power calculation since this study will follow an exploratory approach and generate first effect-size estimators, which can be the basis of the case number estimation for a possible follow-up study. Cross-over effects as well as carry-over effects and period effects will be calculated. For all effects 95% confidence intervals will be given. Since all analyses are of exploratory nature, no adjustment for multiple testing will be made.

### Work package 6

Dealing with data protection law. Basic legal issues regarding data protection, as well as relevant special legal issues regarding data protection (e.g. for workplace monitoring) will be analysed in detail for the use of webcams in neonatal intensive care units. Here, video surveillance, which is generally a relevant data protection process [23], will take place in a particularly sensitive environment that needs a high degree of protection [24]. In addition to the monitored newborns, parents and third parties – such as nurses, doctors and visitors – might also be seen through the camera. Therefore, the legally compliant use of webcams requires the consent of the data subject, or a legal basis for the surveillance (see Art. 6 General Data Protection Regulation (GDPR)). Difficulties in defining the legal basis arise since, in law, data processing in the context of employment (Art. 88 GDPR) has different requirements from data processing outside this particular situation (Art. 6 et seq. GDPR). In the health sector, additional special requirements must be fulfilled (Art. 9 GDPR). However, it is not only the GDPR (the main relevant European data protection framework) that must be taken into account, as national regulations such as the Health Data Protection Act of North Rhine–Westphalia (GDSG NRW) or the Hospital Act of North Rhine–Westphalia (KHG NRW) can also apply. In any case, there is no specific legal basis for the use of video surveillance in hospitals [25]. Therefore, all these legal requirements will be addressed in the study.

## Discussion

To our knowledge, this is the first study that comprehensively accompanies and evaluates the use of webcams on multiple NICUs, with regard to parental outcomes and effects on the medical staff. There are already international studies on the use of webcams on NICUs on the topics of additional workload of the nursing staff [13], attitudes towards the cameras of parents, doctors and nursing staff [14, 26], camera usage behaviour [27] and effects of the camera on the length of stay of the premature baby [28]. However, this study examines for the first time primary and secondary outcomes of parents with respect to camera use using validated scales that allow a direct comparison between intervention and control groups. Furthermore, this study benefits from its high number of cases targeted and the evaluation of effects on both parents and physicians as well as nursing staff. In addition, the compilation of practical guidance in the second year provides the opportunity to counteract preconceptions on the parents’ side concerning the camera use and to inform properly about the camera system.

It should be noted, however, that this study is not a clinical study but a health services research study and therefore evaluates the effectiveness not the efficacy. Methodologically, this results in some limitations of the study. A blinding of the intervention and control group is not possible, since the use of the camera is an obvious intervention which cannot be blinded from any of the persons involved. Furthermore, the measured outcomes are mainly patient reported outcomes reported through questionnaires.

Yet this study can be regarded as a pragmatic trial providing necessary evidence to decide whether to promote the use of webcams on NICUs or not and what to consider when implementing them.

## Data Availability

The anonymous datasets generated during the current study may be made available from the corresponding author on reasonable request. Protocol modifications will be communicated to relevant parties such as the publisher of this study protocol or the trial registry. The study manager will oversee the intra-study sharing process. All project members listed in this study protocol will have access to the cleaned anonymous data sets of their respective work packages.

## Abbreviations

GDPR: General Data Protection Regulation
KHG NRW: Hospital Act of North Rhine–Westphalia
MRC: Medical Research Council
NICU: Neonatal intensive care unit
TDM: Total Design Method
WP: Work packages
